# The Impact of Three White-Rot Fungi on Nutrient Availability, Greenhouse Gas Emissions, and Volatile Fatty Acid Production in Myceliated Sorghum

**DOI:** 10.3390/foods13142199

**Published:** 2024-07-12

**Authors:** Lydia K. Olagunju, Omoanghe S. Isikhuemhen, Peter A. Dele, Felicia N. Anike, Joel O. Alabi, Kelechi A. Ike, Yasmine Shaw, Rosetta M. Brice, Oluteru E. Orimaye, Michael Wuaku, Nkese S. Udombang, Uchenna Y. Anele

**Affiliations:** 1 Department of Animal Sciences, North Carolina Agricultural and Technical State University, 1601 East Market Street, Greensboro, NC 27411, USA; lkolagunju@aggies.ncat.edu (L.K.O.); delepa@funaab.edu.ng (P.A.D.); joalabi@aggies.ncat.edu (J.O.A.); kaike@aggies.ncat.edu (K.A.I.); yashaw@aggies.ncat.edu (Y.S.); rosettab101@gmail.com (R.M.B.); oeorimaye@aggies.ncat.edu (O.E.O.); mwuaku@aggies.ncat.edu (M.W.); uyanele@ncat.edu (U.Y.A.); 2 Department of Natural Resources and Environmental Design, North Carolina Agricultural and Technical State University, 1601 East Market Street, Greensboro, NC 27411, USA; fnanike@ncat.edu (F.N.A.); nsudombang@aggies.ncat.edu (N.S.U.)

**Keywords:** white rot fungi, myceliated sorghum, greenhouse gases, fiber digestibility

## Abstract

Our study employed *Pleurotus ostreatus*, *P. djamor*, and *Trametes versicolor* (white rot fungi = WRF) in the process of solid-state fermentation (SSF) to convert sorghum grains into myceliated sorghum (MS). The MS was then used for in vitro studies to assess changes in nutrient content compared to untreated sorghum (control). The results demonstrated a significant (*p* < 0.001) increase in dry matter (DM), crude protein (CP), ash, neutral detergent fiber (NDF), and acid detergent fiber (ADF) contents of MS. Specifically, CP and ash values saw a remarkable increase from 68 to 330% and 40 to 190% in MS, respectively. Additionally, NDF and ADF degradability values increased significantly (*p* < 0.001) by 81.5% and 56.2% in *P. djamor*-treated MS at 24 h post-incubation. The treatment × time interaction was also significant (*p* < 0.001) for greenhouse gas (GHG) emissions. *T. versicolor* MS exhibited the highest total volatile fatty acid (TVFA) and propionate production. The use of WRF in the SSF process led to a significant improvement in the nutritional value of sorghum. Despite the varying effects of different WRF on the nutritional parameters in MS, they show potential for enhancing the feed value of sorghum in animal feed.

## 1. Introduction

The increasing demand for safe and quality foods is driven by exponential population growth, and a projected to reach 9.15 billion by 2050. To meet this need, meat and milk production is expected to increase by 50–70% [1]. Limited access and high cost of feed resources during the winter pose a serious challenge to sustainable beef production. More so, cattle have been implicated in contributing to about 62% of total greenhouse gas (GHG) emissions [2]. Therefore, animal scientists and cattle farmers seek to address the challenge of increasing cattle production in an environmentally sustainable manner. Cereal grains such as corn, sorghum (milo), wheat, and barley are potential energy sources for beef and dairy cattle. Sorghum compares favorably with corn in terms of energy and has higher CP and ADF content. Due to its structural composition and slower fermentation rate, sorghum has the least tendency to induce ruminal acidosis [3]. Hence, sorghum is the second most used grain as feedstuff for cattle production [4]. However, sorghum has the least nutrient availability when fed to ruminants compared to other grains such as wheat, corn, oats, and barley [3]. It is important to enhance sorghum’s nutrient availability (nutritional value) for its full exploitation in animal feed. Therefore, sorghum grain is often pretreated with steam-flaking, dry rolling, reconstitution, high moisture, popping, exploding, roasting, or micronizing before it can be used in feeding ruminants [4,5]. But the cost associated with pretreatment rendered sorghum an underutilized grain compared to other cereal grains [4,5]. Therefore, strategies to improve nutrient availability in sorghum at a reduced cost will provide a crucial solution in feeding livestock for global food security [6]. Consequently, accomplishing the significance attributed to sorghum to foster sustainable agricultural practices and rural development [5].

Globally, cultivation of edible mushrooms has increased tremendously in the last few decades. China is the largest mushroom producer, growing over 29 million tons, accounting for over 80% of the world’s mushroom production as of 2015 [7]. WRF can grow directly on grains and crop residues to produce myceliated grains and spent mushroom substrates (SMS). They can degrade complex lignocellulose structures because of their high enzymatic capacity, thereby improving the nutritional value of low-quality feed resources.

The edible species of fungi include *Pleurotus*, *Ganoderma*, *Lentinula*, *Lentinus*, *Volvariella*, and *Agaricus* spp., among others. However, the mushroom species commonly cultivated are the *Agaricus bisporus*, *Lentinula edodes*, and *Pleurotus* spp. [8]. However, different fungi strains could exhibit significant variation in ligninolytic, cellulolytic, and hemicellulolytic degradation depending on substrate nutrient composition and fermentation conditions [9,10], as previously stated by the authors of [11], who reported that WRF have different modes of attack.

Previously, Akinfemi et al. [12] reported improvement in nutritive value, gas production, and organic matter digestibility of maize husk treated with four different fungi (*Lentinus subnudus*, *Pleurotus tuberregium*, *P. sajor-caju*, and *P. pulmonarius*) compared with the control group. Additionally, Wang et al. [10] reported improvement in acid detergent lignin (ADL) degradation, CP, and amino acid contents in corn stover treated with four WRF strains (*Pleurotus djamor*, *P. eryngii*, *P. sajor-cajun*, *P. citrinopileatus*). Therefore, the study hypothesized that the treatment of sorghum with different WRF strains will impact the nutritive value of sorghum and enhance its usage for animal feed. Hence, the study aimed to compare the efficacy of three WRF strains on nutrient availability, volatile fatty acids production, and greenhouse gas emissions of sorghum.

## 2. Materials and Methods

### 2.1. Solid-State Fermentation

Spawn was prepared following a modified method previously reported [13]. Briefly, 16 replicate bags weighing 2 kg were prepared using sorghum after soaking them in water for 12 h and drained before sterilization (121 °C, 15 psi for 1 h). Four bags per WRF strain were inoculated with *T. versicolor*, *P. djamor*, and *P. ostreatus* and regarded as Trt 1, Trt 2, and Trt 3, respectively. All the inoculated bags were incubated at 25 °C for 21 days and collectively referred to as myceliated sorghum. After incubation, the bags were dried at 60 °C for 48 h. Four out of the sixteen bags not inoculated were also dried as above and used as control. The MS and the control were milled to pass through a 2 mm sieve size of Retsch miller (model SM 100; Retsch GmbH, Haan, Germany) before being used for in vitro studies.

### 2.2. Animal Care and Feeding

The North Carolina Agricultural and Technical State University Institutional Animal Care and Use Committee (IACUC) approved all animal procedures and uses. This study was conducted at the North Carolina Agricultural and Technical State University Dairy Research and Training Facility (NCAT DRFT; Greensboro, NC, USA). The cannulated cows were observed daily for health problems and treated according to routine management practices at the DRFT maintained under IACUC-approved protocol LA21-009.

### 2.3. Experimental Design

The experiment employed a 3 (incubation time) × 4 (treatments) factorial design with two separate runs. The treatments were untreated, *T. versicolor*, *P. djamor*, and *P. ostreatus*-treated sorghum, named Control, Trt 1, Trt 2, and Trt 3, respectively. A total of three replicates were prepared for each treatment for the various periods. The treatments were evaluated for in vitro dry matter digestibility (IVDMD), the efficiency of the microbial population (PF, partitioning factor), total gas production, volatile fatty acid production, and greenhouse gases. 

### 2.4. Sample Preparation

Treated or untreated (control) sorghum samples obtained from Mushroom Biology and Fungal Biotechnology Laboratory (MBFBL) NCAT farm, approximately 0.5 g each were weighed with an analytical scale (model VWR-224AC; VWR International, Radnor, PA, USA) directly into 100 mL serum bottles (Cat# 223747; Wheaton Science Products, Milville, NJ, USA). Three replicates (bottles) were prepared for each treatment and incubated for 3, 6, and 24 h. To determine digestibility, 0.5 g of the samples were weighed into Ankom bags (57; Ankom Technology Corp, Macedon, NY, USA), sealed using a heat impulse sealer MP-8 Intertek (Midwest Pacific, CA, USA), and inserted into pre-labeled 100 mL serum bottles according to treatment with three replicates per treatment.

### 2.5. In Vitro Batch Culture

A detailed description of the batch culture study was described in [14,15]. The inoculum was sampled from two ruminally cannulated grazing Holstein cows. The animals were fed 18% protein grain, corn silage, and alfalfa hay daily and had unrestricted access to clean drinking water. The inoculum was mixed with artificial saliva [16] in a 1:3 ratio (15 mL of inoculum: 45 mL of artificial saliva) and incubated at 39 °C for 3, 6, and 24 h. Using a pressure transducer, the accumulated headspace gas pressure was measured at each of the three times points. Blanks were included to correct the produced gas from the buffered inoculum. Corrected gas pressure values were used to estimate gas production [17]. 

### 2.6. Estimation of Greenhouse Gases

Ammonia (NH_3_), carbon dioxide (CO_2_), hydrogen sulfide (H_2_S), and CH_4_ concentrations were estimated using a portable gas analyzer (Biogas 5000, Landtec, Dexter, MI, USA). The gas analyzer was calibrated per the manufacturer’s instructions. An aliquot of gas from the samples was introduced into the analyzer with a 22 mm gauge needle attached to the end of the inlet Tygon tube. The unit was purged between each sampling to eliminate any residual gas from the previous sampling.

### 2.7. In Vitro Dry Matter Digestibility

After gas readings, the Ankom bags were removed from the bottles, rinsed, and dried in a 55 °C oven for 48 h. In vitro apparent degradable dry matter (IVADDM) and in vitro true degradable dry matter (IVTDDM) were estimated as described in [18].

### 2.8. Laboratory Analysis

#### Chemical Analysis

The dietary samples were dried in a forced-air oven at 55 °C for 48 h to determine DM using method #930.15 [19]. Petroleum ether was used to determine the ether extract (EE) content using an Ankom XT15 fat analysis system (Ankom Technology Corp., Fairport, NY, USA) (EE; #920.39) according to AOAC (2000) [20]. The ash content of the samples was measured via the procedure described in [21]. The organic matter (OM) was calculated by subtracting the ash content from 100. The samples were analyzed for their nitrogen content via the Pregl–Dumas method. The crude protein (CP) content was estimated by multiplying the nitrogen value by 6.25. The procedure described in [22] was used to analyze the NDF content using heat-stable amylase with sodium sulfite, and the acid detergent fiber (ADF; [23]) content was analyzed sequentially, using the same bags as the NDF analysis with an Ankom 200 Fiber Analyzer (Ankom, Macedon, NY, USA). The acid detergent lignin (ADL) content was determined by soaking in 72% sulfuric acid (H_2_SO_4_) based on ANKOM Technologies analytical methods. The hemicellulose content was calculated using NDF-ADF, and the cellulose content was calculated using ADF-ADL.

### 2.9. Microbial Mass Estimation

Microbial mass (Mmass) was determined according to the protocol described in [22,24]. The pellet samples were removed from the freezer and decapped before they were arranged in aluminum pans. The samples were lyophilized for 96 h using a BUCHI freeze-dryer (model DUO 6 M; BUCHI Labortechnik AG, Flawil, Switzerland). The lyophilized samples were weighed to determine the weight of the pellets, and the Mmass was calculated. The partitioning factor (PF) was estimated as the ratio of mg of substrate truly degraded/mL of gas produced.

#### 2.9.1. Volatile Fatty Acid

The preserved rumen fluid samples were thawed and centrifuged at 10,000× *g* for 15 min at 4 °C, and the volatile fatty acid (VFA) concentration was analyzed. Gas chromatography with Flame Ionization Detection (FID) was used to quantify the VFA concentration, and a metaphosphoric–crotonic acid mixture was used as an internal standard. The sample injection volume was set at 1 μL while maintaining a split ratio of 1:12. The injector port was kept at a constant temperature of 250 °C. Helium was used as the carrier gas at a flow rate of 1 mL/min, facilitating the efficient transport of the sample through the GC column. A temperature gradient was employed in the oven to optimize the separation of the analytes. Initially, the oven temperature was set at 120 °C for 0.8 min, followed by a controlled increase of 8 degrees per minute until 140 °C was reached. The oven was maintained at 140 °C for 1.8 min. The detector temperature was maintained at 280 °C. The FID operation was supported by a controlled flow of hydrogen and air gases with 30 mL/min flow rates and 400 mL/min, respectively. Additionally, N was used as a make-up gas at a 25 mL/min flow rate, ensuring a stable baseline and consistent detector performance.

#### 2.9.2. Statistical Analysis

Data on nutrient degradability, GHG production, fermentation parameters, and VFA concentration were analyzed using One-way Analysis of Variance [25]. Significant means were separated at *p* ≤ 0.05 using the Duncan multiple range test.

## 3. Results

### 3.1. Chemical Composition of Treated and Untreated Sorghum

The chemical composition of untreated and MS grain is shown in Table 1. Higher (*p* < 0.001) DM was obtained in treated sorghum samples compared to control. The organic matter content was significantly higher (*p* < 0.001) in Trt 1 compared to other groups. CP and ash contents increased in WRF-treated sorghum. MS samples’ CP significantly (*p* < 0.001) increased by 68, 215, and 330% while ash content increased by 40, 127, and 190% in Trt 1, Trt 2, and Trt 3, respectively. Ether extract (EE) content was reduced in MS from approximately 32 to 41% compared to untreated sorghum. Trt 3 had the lowest (*p* < 0.001) EE content. The highest (*p* < 0.001) NDF value was observed in untreated sorghum, while Trt 2 had the lowest value. Trt 1 had the highest (*p* < 0.001) ADF compared to other groups. Among the MS, higher (*p* < 0.001) ADL was obtained in Trt 2 (13.27%) and Trt 3 (12.64%), while Trt 1 had the lowest value (3.79%). The hemicellulose content was higher (*p* < 0.001) in control and Trt 3 compared to Trt 1 and Trt 2. The cellulose content was higher (*p* < 0.001) in Trt 1 and untreated sorghum than Trt 2 and Trt 3.

### 3.2. Dry Matter and Fiber Fractions Degradability 

The effects of the three WRF treatments on DM, NDF, ADF, and ADL degradability are presented in Table 2. Trt × time interaction significantly (*p* < 0.005) influenced the DM, NDF, ADF, and ADL degradability. *P. djamor* had significantly (*p* < 0.001) lower DM degradability values than other treatments. *P. djamor* had significantly (*p* < 0.001) higher NDF, ADF, and ADL degradability among the treated MS followed by *P. ostreatus* and *T. versicolor*. *P. djamor*-treated sorghum increased in NDF degradability by 75.5, 76.7, and 81.5% compared to untreated sorghum at 3, 6, and 24 h, respectively. Additionally, *P. djamor*-treated sorghum resulted in higher ADF degradability by 62.7 and 62.9% compared to *T. versicolor*-treated MS at 3 and 6 h post-incubation and was 56.2% higher at 24 h compared to untreated sorghum. Treating sorghum with *P. djamor* showed greater (*p* < 0.001) ADL digestibility by 95.8, 93.2, and 88.4% compared with sorghum treated with *T. vesicolor* at 3, 6, and 24 h post-incubation, respectively.

### 3.3. Greenhouse Gases Production 

The main and interaction effects of mushroom strain and time significantly (*p* < 0.001) influenced total gas volume and greenhouse gas (methane, carbon dioxide, ammonia, and hydrogen sulfide) production, as presented in Table 3. Trt × time interaction significantly (*p* < 0.005) influenced the total gas production and GHG emissions. Untreated sorghum produced less (*p* < 0.001) GHG and total gas volume until 6 h but was significantly higher than sorghum treated with *T. versicolor* and *P. djamor* at 24 h post-incubation. Among the treated groups, methane and carbon dioxide emissions were lower (*p* < 0.001) in *P. djamor* at 3 h (64 and 66%) and 6 h (46.8 and 66.1%) post-incubation compared to *P. ostreatus*. At 24 h post-incubation, the total gas production and GHG emissions in sorghum treated with *P. djamor* significantly (*p* < 0.001) decreased by 29 to 32% and 33 to 44% compared with control and *T. versicolor*-treated samples.

### 3.4. In Vitro Digestibility and Fermentation Parameters

The effects of the different WRF-treated sorghum on undegraded DM, in vitro apparent degradable dry matter (IVADDM), in vitro true degradable dry matter (IVTDDM), partitioning factor (PF), short-chain fatty acid (SCFA) production and microbial mass are presented in Table 4. Trt × time interaction significantly (*p* < 0.005) influenced the DM, IVADDM, IVTDDM, PF, and microbial mass. Undegraded DM values differed (*p* < 0.001) among all treatments throughout incubation. *P. djamor*-treated sorghum had higher undegraded DM compared with untreated sorghum. There was a significant reduction (*p* < 0.001) in the IVTDDM value for sorghum treated with *P. djamor* throughout the fermentation period compared with other treatments. The partitioning factor (PF) reported at 24 h post-incubation showed a reduced value (*p* < 0.05) in *P. djamor* when compared with *P. ostreatus* treated and untreated sorghum. The SCFA was significantly influenced (*p* < 0.001) by Trt and incubation time. Expectedly, SCFA significantly (*p* < 0.001) increased with incubation time. Significant (*p* < 0.001) differences existed in microbial mass values across the treatments and over time. The microbial mass of sorghum treated with *T. versicolor* was similar to the untreated sorghum, while sorghum treated with *P. djamor* had the lowest (*p* < 0.001) values throughout the incubation time.

### 3.5. Volatile Fatty Acids Production

The effects of treatments and incubation time on the total and molar proportions of volatile fatty acid production (TVFA) are presented in Table 5. Trt × time interaction significantly (*p* < 0.005) influenced the TVFA, acetate, propionate, butyrate, and acetate: propionate ratio (APR). *T. versicolor*-treated sorghum and control had the highest (*p* < 0.001) TVFA and propionate concentration at 24 h of incubation. The acetate concentration increased by 10%, whereas propionate reduced by about 19–21% at 24 h of incubation in sorghum treated with *P. djamor* and *P. ostreatus* compared with the untreated sorghum. TVFA production increased with incubation time. The molar proportion of acetate decreased while propionate and butyrate increased between 6 h and 24 h of incubation. Consequently, APR decreased with incubation time.

## 4. Discussion

The chemical analysis results revealed significant improvement in the nutritive value of MS compared with untreated sorghum. This confirmed the report by Benson et al. [26], who noted that the biological properties of raw grain were altered by fungal fermentation, and was also consistent with previous studies [10,14] when Pleurotus mycelium enhanced the feeding value of corn stover. The increase in DM content of MS implies that MS would potentially offer more nutrients when fed to the animals than untreated sorghum, which might have resulted from the release of bound nutrients during the solid-state fermentation [14]. It is generally known that a feedstuff’s DM content depicts various nutrients available to the animal [14]. The increase in CP content of MS that ranged from 68 to 330% compared to untreated sorghum might have resulted from bio-availability of protein [6]. Kafirin protein might have been disrupted within the protein body structure during the myceliation of treated samples, and the addition of protein content from fungi mycelial biomass might have led to increased values of protein content in treated sorghum. Furthermore, kafirins constitute the main seed storage proteins in sorghum, accounting for 70–80% of total protein and majorly contributing to the poor digestibility of sorghum grain. Sorghum kafirins are sub-classified into α-, β-, γ-, and δ-kafirin. α-kafirin accounted for 80% of kafirin protein content, which is encapsulated at the center of the spherical protein body by β- and γ-kafirin with a small amount of δ-kafirin. Particularly, α-kafirins are resistant to digestion because of their location. Additionally, inter- and intra-molecular disulfide bonds between β- and γ-kafirin proteins lead to a high degree of protease resistance in protein bodies. Therefore, digestibility is affected by the tight matrix around starch granules and endosperm protein. Hence, the protein outer layer might have been altered during colonization by WRF, which acted as a biological pretreatment. This might have provided more access for proteinases to digest α-kafirin and resulted in higher digestibility of sorghum grain [27]. The mycelium colonization and degradation could have enhanced the bioavailability and digestion of protein in the myceliated substrates. The higher CP content of MS indicates its adequacy to contribute more protein to meet the needs of rumen microbes and cattle when combined with low CP forages to formulate a total mixed ration (TMR). 

According to Duodu et al. [28], low protein digestibility of sorghum is linked to the tough inner protein wall surrounding the seed, the protein (karifin proteins) crosslinking, as well as the interaction of sorghum proteins within themselves and with non-protein components which impede amino acids to proteolysis; however [6], this is attributed the low protein digestibility to the presence of anti-nutritional factors. Meanwhile, solid-state fermentation has been reported to increase the protein and amino acid concentrations of substrates or products [26,29]. In agreement, Kim et al. [30] reported higher CP contents in spent *Agaricus bisporus* substrates. Akinfemi et al. [12] also observed an increase in CP levels from 7.44 to 9.90% in maize husk treated with four strains of fungi (*L. subnudus*, *P. tuber-regium*, *P. sajor-caju*, and *P. pulmonarius*). 

The ash content of MS in this study increased by 40, 127, and 190% in sorghum treated with *T. versicolor*, *P. djamor*, and *P. ostreatus*, respectively, compared with untreated sorghum. High ash content indicated improved mineral availability. This agrees with the findings of the authors of [12], who reported a more than a three-fold increase in ash content of maize husk treated with *P. pulmonarius* against the control. Previous studies have reported the high ash content and an appreciable amount of major and trace minerals in spent mushroom substrates suitable for feeding livestock [31].

Fiber constitutes a crucial nutrient in the ruminant diet for proper rumen function and overall animal health. However, the capacity for fiber degradation to produce the primary energy source for ruminants varies depending on the different nutrients in the diet.

In this study, all three white-rot fungi (WRF) induced substantial alterations in the neutral detergent fiber (NDF), acid detergent fiber (ADF), and acid detergent lignin (ADL) content of MS. Lower NDF values were observed in the three WRF-treated sorghum compared to untreated sorghum, indicating increased voluntary intake when fed to the animals. Higher ADF and cellulose, along with lower ADL levels observed in samples treated with Trametes versicolor, suggest improved feed quality and digestibility. Consequently, this may enhance the availability of total digestible nutrients for animals. It has been reported that cellulose converts to glucose during ruminal degradation, which, in turn, is transformed into energy [32]. Improved digestibility that resulted in decreased acetate production at 3 h in treated samples compared to untreated sorghum suggested a shift in the rumen fermentation pathway that indicated the production of more glucose from the availability of more nutrients, and, consequently, the production of more energy during ruminal degradation. Higher lignin content in *P. djamor* and *P. ostreatus* treatments could be related to their lower abilities to degrade lignocellulose complex compared to *T. versicolor*, as well as the presence of fungi mycelia and associated macromolecules in the substrates [14]. Also, Mishra et al. [9] reported that WRF strains have different ligninolytic, cellulolytic, and hemicellulolytic abilities and often demonstrate varying enzymatic hydrolysis during solid-state fermentation. 

Solid-state fermentation process and microbial technology have been employed for cost-effective, large-scale production of single or cocktail of enzymes not only for use in the animal feed industry but also in the food and beverage, biofuel, cosmetic, detergent, fabric, and pharmaceutical industries [29]. It is known that nutrient contents of various crops and agro-industrial by-products can be improved with mono- and co-cultures of yeasts, bacteria, and filamentous fungi [29]. It has been noted that the mycelial action of mushrooms produces a myriad of extra-cellular enzymes such as laccase, peroxidase, β-glucosidase, xylanase, and cellulase, which help in fiber and lignocellulosic degradation. Therefore, significant variations observed in this study on the nutritive value of myceliated treated sorghum with the three WRF could be attributed to the differences in the type and quantity of enzymes produced by the fungi strains [8]. The values obtained for *P. djamor* and *P. ostreatus* are expected because both are gilled mushrooms and are the same genus, which is significantly different from *T. versicolor*, a polypore. 

High DM disappearance in sorghum treated with *T. versicolor* and *P. ostreatus* was similar to untreated sorghum, which resulted in lower undegraded portions, thus implying better nutrient availability [14]. Higher NDF, ADF, and ADL degradability in MS compared to untreated sorghum indicates that the cell wall components are being greatly degraded by enzymes secreted during solid-state fermentation [9,30]. Increased DM disappearance noted for MS would likely increase soluble polysaccharide availability and digestion when fed beef and dairy cattle. The NDF digestibility is positively correlated with the total digestible nutrients (TDN). This may imply that an increase in the NDF content of the feedstuff or total mixed ration would increase the TDN content and, consequently, dietary energy content [14]. Higher NDF, ADF, and ADL degradability were obtained in sorghum treated with *P. djamor.* This indicates that WRF does vary in their biomodification abilities, and screening WRF strains to select the best for nutrient improvement in sorghum to be used in animal feed is critical. Studies [8,10,31] have reported a significant effect of WRF on fiber degradability and ruminal digestibility of spent substrates. 

Among the WRF investigated in this study, *T. vesicolor* proved to be more effective at reducing the undegradable sorghum fraction than *P. djamor* and *P. ostreatus*. More so, higher IVTDDM in MS treated with *T. versicolor* might be linked to reduced ADL degradability. The PF depicts the partitioning of truly digested organic matter between microbial biomass and fermentation gases during fermentation; hence, arithmetically, the PF is inversely related to gas volume [33]. In this study, the PF value was lower in *P. djamor* compared to *P. ostreatus* and untreated samples at 24 h post-incubation. The lower microbial mass obtained in MS treated with *P. djamor* could be connected to a higher undegraded portion, decreased gas volume, and suppressed in vitro true dry matter digestibility compared to other groups. Brice et al. [34] confirmed higher microbial mass enhances the efficiency of microbial protein synthesis, thereby making more nutrients available from the digest.

High-fibrous feed with low digestibility causes higher energy loss from dietary intake and increases GHG emissions [35]. Most methane from agriculture arises primarily from enteric fermentation, and ruminants are majorly implicated in emitting CH_4_ gas [2]. The GHG (CH_4_, CO_2_, NH_3,_ and H_2_S) emissions were considerably lower in MS treated with *P. djamor*. In this study, GHG emissions in sorghum treated with *P. djamor* significantly reduced by 66% at 3 h post-incubation compared to *P. ostreatus*. The reduction at 24 h post-incubation was 33–44% compared with sorghum treated with *T. versicolor*. This implies that solid-state fermentation by different fungi strains could influence ruminal fermentation, nutrient digestion, and gas production differently. Hence, this is very significant and crucial since methane production is an energy loss to the animal during ruminal digestion because methanogenesis results in a loss of 2–12% gross energy, which is responsible for nearly 5% of the global anthropogenic GHG emissions [2,35]. Therefore, minimal CH_4_ would result in a higher energy reserve for the ruminant animals, improve feed efficiency, and reduce negative environmental impacts [34]. Thus, *P. djamor* can be recommended for solid-state fermentation of sorghum to minimize GHG production and climate change effects.

The higher gas volume noted in MS at 3 h could be due to the carbohydrate solubilizing enzymes released by WRF to saccharify complex sugars (starch, cellulose, and hemicellulose) in the substrates, thereby making cellulose more readily accessible to the rumen microbes for fermentation. Gas production depicts the accessibility of degradable carbohydrates, particularly cellulose, for enteric fermentation and is positively correlated to VFA production. Consequently, rumen epithelium can absorb VFA, a major energy source for ruminant animals [35]. At 24 h incubation, higher gas production is positively related to higher TVFA. Gas production significantly varied, with *T. versicolor* having the highest gas production compared to other treatments. The different gas volume production from MS samples suggests reduced feed residence in the rumen, which indicated that MS was significantly influenced by the WRF strains with the ability to release more TVFA when compared with the untreated sorghum due to its slow rate of digestion and longer residence in the rumen. Akinfemi et al. [12] also reported that *L. subnudus*, *P. tuber-regium*, *P. sajor-caju* and *P. pulmonarius* improved gas production in treated maize husk substrate when compared with the untreated group, thus resulting in better digestibility, although *L. subnudus* produced more gas than *P. tuber-regium*. The increased digestibility with increased gas production would increase the animal’s main energy sources and anabolic precursors. The numerical increase in TVFA and molar proportion of propionate production at 3 h and 6 h post-incubation indicated improved digestibility for MS when compared with untreated sorghum due to its slow rate of digestion. The study showed that the rumen microbes would degrade more myceliated substrate within a shorter period, positively impacting the fermentation pathway that favors improved energy availability for animal production. More so, increased propionate formation in the rumen acts as an alternative H_2_ sink through the succinate and acrylate pathways, thereby reducing methanogenesis [35], while higher acetate is linked with increased methane concentration [34]. 

Solid-state fermentation significantly reduced methane production at 24 h post-incubation in *Pleurotus djamor*-treated samples compared to untreated sorghum. This can be related to better digestibility and propionate pathway, which might have initiated H_2_ sink, thereby reducing methanogenesis during enteric fermentation. Furthermore, reduced methane emission means more energy is retained and nutrient availability is increased during digestion. The higher ammonia concentration observed from the solid-state fermented sorghum samples confirmed the increased crude protein levels in the treated samples and increased protein digestibility. Therefore, the myceliation of sorghum with white rot fungi could provide cost-effective biotechnology for its transformation to protein-rich feed [36]. Protein-rich feed enhances carbohydrate digestion and optimum microbial protein synthesis in the rumen [37]. The higher protein in myceliated sorghum (due to solid-state fermentation) could provide dietary CP to fulfill the amino acid requirements for animal growth, maintenance, optimal health, and reproduction. Protein has been reported as the most expensive ration component in cattle production [38]. Though the myceliated sorghum reduced methane emissions and probably animal production costs, the trade-off in terms of cost due to solid-state fermentation needed to upgrade the sorghum requires further research.

## 5. Conclusions

Overall, the solid-state fermentation of sorghum grain with WRF improved the feeding quality and positively impacted the nutritional value of treated sorghum. The WRF enhanced DM, rumination fiber components, and the treated sorghum’s fiber degradability. However, there were variations in the outcome among the three strains investigated. *T. versicolor*-treated sorghum produced higher IVTDDM, TVFA, and propionate concentrations than the other strains with an improved voluntary intake compared to the untreated sorghum. This suggests that animals will consume more of *T. versicolor*-treated sorghum, thereby resulting in more nutrients for better performance and production. However, *P. djamor* reduced the production of GHG, which indicated that the increased fibrous components from the solid-state fermentation are readily digestible fiber and will not induce the acetate pathway during ruminal fermentation; therefore, energy loss would be minimized. This study revealed that WRF myceliated sorghum could be an improved feed material for animal feed.

## Figures and Tables

**Table 1:** Chemical composition (% dry matter) of untreated and myceliated sorghum *.

	Control	Trt 1	Trt 2	Trt 3	SEM	*p*-Value
Dry matter	88.76 ^d^	92.45 ^b^	91.36 ^c^	92.96 ^a^	0.29	<0.001
Organic matter	87.45 ^b^	90.25 ^a^	87.23 ^d^	87.33 ^c^	0.23	<0.001
Crude protein	6.75 ^d^	9.44 ^c^	15.31 ^b^	19.56 ^a^	0.90	<0.001
Ether extract	13.76 ^a^	9.36 ^b^	9.30 ^b^	8.08 ^c^	0.39	<0.001
Ash	1.31 ^d^	2.20 ^c^	4.13 ^b^	5.63 ^a^	0.30	<0.001
NDF	69.55 ^a^	56.21 ^b^	46.30 ^c^	60.62 ^b^	1.78	<0.001
ADF	20.07 ^b^	26.13 ^a^	15.11 ^c^	15.45 ^c^	0.83	<0.001
ADL	3.02 ^b^	3.79 ^b^	13.76 ^a^	12.64 ^a^	0.79	<0.001
Hemicellulose	49.48 ^a^	30.08 ^b^	31.19 ^b^	45.17 ^a^	1.90	<0.001
Cellulose	17.05 ^b^	22.33 ^a^	2.31 ^c^	2.81 ^c^	1.47	<0.001

* *n* = 8 replicates; NDF, neutral detergent fiber; ADF, acid detergent fiber; ADL, acid detergent lignin; SEM, standard error of means; ^a–d^ means with different superscripts within the same column differ, *p* < 0.05.

**Table 2:** Dry matter and fiber fractions degradability of untreated and myceliated sorghum.

Time	Trt	DMD (%)	NDFD (%)	ADFD (%)	ADLD (%)
3 h	Control	17.71 ^def^	12.42 ^f^	30.89 ^c^	3.18 ^b^
	Trt 1	19.97 ^cde^	14.34 ^f^	23.75 ^d^	1.57 ^d^
	Trt 2	10.93 ^f^	50.78 ^c^	63.73 ^a^	37.33 ^a^
	Trt 3	24.54 ^bcd^	44.05 ^d^	53.77 ^b^	9.77 ^bc^
6 h	Control	19.17 ^cde^	12.82 ^f^	29.84 ^c^	3.62 ^cd^
	Trt 1	14.55 ^ef^	19.15 ^e^	23.94 ^d^	2.36 ^cd^
	Trt 2	10.09 ^c^	55.08 ^b^	64.49 ^a^	34.75 ^a^
	Trt 3	31.42 ^b^	43.71 ^d^	52.44 ^b^	2.63 ^cd^
24 h	Control	49.38 ^a^	12.05 ^f^	28.92 ^c^	12.79 ^b^
	Trt 1	53.09 ^a^	20.93 ^e^	29.82 ^c^	4.53 ^cd^
	Trt 2	27.04 ^bc^	65.24 ^a^	66.01 ^a^	39.10 ^a^
	Trt 3	51.34 ^a^	48.17 ^c^	56.74 ^b^	4.65 ^cd^
SEM		1.91	2.27	1.99	2.23
Trt		<0.001	<0.001	<0.001	<0.001
Time		<0.001	<0.001	0.0338	<0.001
Trt × Time		0.0015	<0.001	0.2122	<0.001

DMD, dry matter degradability; NDFD, neutral detergent fiber degradability; ADFD, acid detergent fiber degradability; ADLD, acid detergent lignin degradability; Trt, treatment; SEM, standard error of means; ^a–f^ means with different superscripts within the same column differ, *p* < 0.05.

**Table 3:** Total gas production, methane, carbon dioxide, ammonia, and hydrogen sulfide concentration of untreated and myceliated sorghum.

Time	Trt	Gas Volume (mL/g DM)	Methane (mg/g DM)	Carbon Dioxide (mg/g DM)	Ammonia (mmol/g DM)	Hydrogen Sulfide (mg/g DM)
3 h	Control	3.14 ^i^	0.015 ^c^	0.84 ^i^	1.17 ^c^	5.82 ^d^
	Trt 1	9.62 ^hi^	0.056 ^c^	2.79 ^hi^	6.23 ^c^	25.23 ^d^
	Trt 2	9.39 ^hi^	0.048 ^c^	2.60 ^hi^	6.01 ^c^	25.22 ^d^
	Trt 3	12.94 ^h^	0.075 ^c^	3.94 ^hi^	7.82 ^c^	33.04 ^d^
6 h	Control	21.68 ^g^	0.178 ^c^	5.75 ^gh^	29.23 ^c^	104.97 ^d^
	Trt 1	31.14 ^f^	0.347 ^c^	9.44 ^f^	46.95 ^c^	190.00 ^d^
	Trt 2	26.76 ^fg^	0.259 ^c^	8.36 ^fg^	36.51 ^c^	148.95 ^d^
	Trt 3	40.13 ^e^	0.554 ^c^	12.75 ^e^	58.34 ^c^	245.01 ^d^
24 h	Control	144.44 ^b^	5.402 ^a^	47.82 ^b^	316.32 ^ab^	1360.19 ^c^
	Trt 1	160.79 ^a^	5.565 ^a^	52.30 ^a^	388.90 ^a^	2165.38 ^a^
	Trt 2	102.42 ^d^	3.741 ^b^	32.71 ^d^	216.08 ^b^	1246.35 ^c^
	Trt 3	128.22 ^c^	5.206 ^a^	40.59 ^c^	297.97 ^ab^	1813.65 ^b^
SEM		6.73	0.30	2.21	18.18	95.17
Trt		<0.001	<0.001	<0.001	0.3138	0.0120
Time		<0.001	<0.001	<0.001	<0.001	<0.001
Trt × Time		<0.001	<0.001	<0.001	0.0301	0.0096

Trt, treatment; SEM, standard error of means; ^a–i^ means with different superscripts within the same column differ, *p* < 0.05.

**Table 4:** Effects of three WRF-treated and untreated sorghum on some in vitro rumen fermentation parameters.

Time	Trt	Undegraded	IVADDM	IVTDDM	PF	SCFA	Mmass
3 h	Control	0.046 ^f^	0.337 ^ab^	0.900 ^b^		0.015 ^i^	0.258 ^ab^
	Trt 1	0.060 ^f^	0.369 ^a^	0.879 ^b^		0.170 ^hi^	0.251 ^b^
	Trt 2	0.220 ^a^	0.350 ^ab^	0.541 ^f^		0.164 ^hi^	0.092 ^e^
	Trt 3	0.160 ^b^	0.356 ^ab^	0.673 ^e^		0.249 ^h^	0.156 ^d^
6 h	Control	0.048 ^f^	0.356 ^ab^	0.897 ^b^		0.458 ^g^	0.251 ^b^
	Trt 1	0.078 ^e^	0.311 ^b^	0.840 ^c^		0.684 ^f^	0.257 ^ab^
	Trt 2	0.227 ^a^	0.311 ^b^	0.521 ^f^		0.580 ^fg^	0.099 ^e^
	Trt 3	0.141 ^c^	0.343 ^ab^	0.710 ^e^		0.889 ^e^	0.177 ^d^
24 h	Control	0.026 ^g^	0.344 ^ab^	0.945 ^a^	3.10 ^a^	3.392 ^b^	0.283 ^a^
	Trt 1	0.050 ^f^	0.342 ^ab^	0.900 ^b^	2.79 ^ab^	3.783 ^a^	0.277 ^ab^
	Trt 2	0.212 ^a^	0.334 ^ab^	0.549 ^f^	2.56 ^b^	2.388 ^d^	0.101 ^e^
	Trt 3	0.116 ^d^	0.321 ^ab^	0.773 ^d^	2.98 ^a^	3.004 ^c^	0.222 ^c^
SEM		0.008	0.005	0.018	0.07	0.161	0.009
Trt		<0.001	0.7758	<0.001	0.0318	0.7421	<0.001
Time		0.4740	0.1074	0.4863	-	<0.001	0.3028
Trt × Time		<0.001	0.0428	<0.001	-	<0.001	<0.001

IVADDM, in vitro apparent degradable dry matter; IVTDDM, in vitro true degradable dry matter; PF, partitioning factor; SCFA, short chain fatty acid; Mmass, microbial mass; Trt, treatment; SEM, standard error of means; ^a–i^ means with different superscripts within the same column differ, *p* < 0.05.

**Table 5:** Effects of three WRF-treated and untreated sorghum on total and molar proportion of VFA production.

Time	Trt	TVFA	Acetate	Propionate	Butyrate	Iso-Butyrate	Valerate	Iso-Valerate	Acetate: Propionate
3 h	Control	54.16 ^d^	0.737 ^a^	0.164 ^d^	0.082 ^c^	0.00432	0.0114	0.00183	4.51 ^a^
	Trt 1	56.61 ^cd^	0.726 ^a^	0.170 ^d^	0.086 ^bc^	0.00075	0.0110	0.00172	4.27 ^ab^
	Trt 2	57.41 ^cd^	0.733 ^a^	0.166 ^d^	0.084 ^bc^	0.00440	0.0112	0.00184	4.44 ^a^
	Trt 3	56.04 ^d^	0.710 ^a^	0.181 ^cd^	0.090 ^bc^	0.00490	0.0121	0.00204	3.94 ^b^
6 h	Control	61.05 ^cd^	0.720 ^a^	0.174 ^d^	0.088 ^bc^	0.00483	0.0115	0.00187	4.14 ^ab^
	Trt 1	62.08 ^cd^	0.720 ^a^	0.174 ^d^	0.089 ^bc^	0.00421	0.0111	0.00168	4.16 ^ab^
	Trt 2	62.98 ^cd^	0.722 ^a^	0.173 ^d^	0.088 ^bc^	0.00429	0.0115	0.00178	4.18 ^ab^
	Trt 3	65.91 ^c^	0.710 ^a^	0.180 ^cd^	0.091 ^bc^	0.00418	0.0126	0.00179	3.96 ^b^
24 h	Control	106.29 ^a^	0.618 ^c^	0.247 ^a^	0.118 ^a^	0.00396	0.0121	0.00182	2.53 ^d^
	Trt 1	107.56 ^a^	0.624 ^c^	0.241 ^a^	0.117 ^a^	0.00419	0.0118	0.00174	2.62 ^d^
	Trt 2	95.36 ^b^	0.682 ^b^	0.199 ^bc^	0.090 ^abc^	0.00467	0.0124	0.00196	3.45 ^c^
	Trt 3	96.97 ^b^	0.680 ^b^	0.195 ^b^	0.106 ^ab^	0.00423	0.0139	0.00198	3.51 ^c^
SEM		2.54	0.005	0.004	0.002	0.000094	0.00027	0.000062	0.08142
Trt		0.5415	0.0131	0.0021	0.6276	0.8246	0.2502	0.7024	0.0052
Time		<0.001	<0.001	<0.001	<0.001	0.6916	0.2280	0.8324	<0.001
Trt × Time		<0.001	<0.001	<0.001	0.0019	0.7220	0.7400	0.9958	<0.001

Trt, treatment; SEM, standard error of means; ^a–d^ means with different superscripts within the same column differ, *p* < 0.05.

## Data Availability

The original contributions presented in the study are included in the article, further inquiries can be directed to the corresponding author.
